# An integrated humanities–social sciences course in health sciences education: proposed design, effectiveness, and associated factors

**DOI:** 10.1186/s12909-020-02022-7

**Published:** 2020-04-19

**Authors:** Jihyun Lee, Jueyeun Lee, Il Young Jung

**Affiliations:** 1 Department of Dental Education, School of Dentistry, Seoul National University, Seoul, Republic of Korea; 2 Department of Preventive Dentistry, College of Dentistry, Yonsei University, Seoul, Republic of Korea; 3 Department of Conservative Dentistry, College of Dentistry, Yonsei University, Seoul, Republic of Korea

**Keywords:** Medical/Dental humanities, Curriculum, Achievement, Social medicine/dentistry, KASA (knowledge, attitudes, skills, and aspirations)

## Abstract

**Background:**

Previous research has not provided enough direction regarding effective content design of courses integrating the humanities and social sciences in medical and dental education. This study aims at exploring how an Integrated Medical/Dental Humanities–Social Medicine/Dentistry course may be designed; how effective it may be in terms of student growth in knowledge, attitudes, skills, and aspirations; and associated factors.

**Methods:**

The course was designed by distilling commonalities in the international standards for medical/dental education proposed by seven major health organizations. This analysis resulted in a curriculum covering nine major topics: history, professionalism, communication, ethics, management, policy, insurance, law, and research methodology. During the 2017 calendar year, data was collected and statistically analyzed from 68 third-year pre-doctoral students enrolled in the resulting MDHS 13-week course.

**Results:**

Participants showed growth in skills, aspirations, knowledge, and attitudes, with the greatest change occurring in skills, then aspirations, knowledge, and attitudes. Knowledge growth was the only variable significantly related to student achievement of course objectives (*β* = 0.635, *t* (63) = 3.394*, p* = 0.001). The topics that students perceived as most critical were insurance, policy, management, and law. The perceived importance of research was most common among participants and was significantly related to all learning outcomes (For knowledge, *β* = 0.213, *t* (63) = 2.203, *p* = 0.031; for attitudes, *β* = 0.784, *t* (63) = 10.257, *p* = 0.000; for skills, *β* = 0.769, *t* (63) = 9.772, *p* = 0.000; and aspirations *β* = 0.639, *t* (63) = 7.595, *p* = 0.000).

**Conclusions:**

This study proposed a framework for humanities-social sciences education in health sciences education and analyzed its implementation. The empirical evaluation of its effectiveness and factors related to successful outcomes found that students perceived gains in their knowledge, attitudes, skills, and aspirations for humanistic and social aspects of dentistry/medicine. In addition, their recognition of the importance of research was associated with the greatest growth in all four learning outcomes. This study may contribute to the improved design of integrated humanities–social sciences courses.

## Background

Over the last few decades, the field of health sciences education has aimed at producing doctors with scientific knowledge of disease and treatment as well as insight into the personal and societal contexts in which patients’ problems arise. In spite of the long-standing debates and difficulties inherent in such a task, medical and dental education institutions have introduced humanities and social sciences courses to their curricula. Instead of offering separate courses, however, many institutions have established an integrated approach under the rubric of Medical/Dental Humanities-Social Medicine/Dentistry (MDHS) education. MDHS is an interdisciplinary approach to medical/dental education that seeks to incorporate relevant learning experiences in the humanities and social sciences into medicine and dentistry [1–4]. The backdrop to this change is the rising global demand for health care and welfare, and the belief that future medical practitioners with a solid understanding of the humanistic and social aspects of medicine will make greater contributions to human health and life [5, 6].

In integrating the humanities and social sciences, MDHS education may achieve more persuasive rationale and broader contextualization [5]. Studies on medical education have described the future roles of doctors in light of changes in healthcare and society. The reports *Tomorrow’s Doctors* by the General Medical Council (GMC) [7], *The Role of the Doctor* by the World Federation for Medical Education (WFME) [8], and the *Five Star Doctor* by the World Health Organization (WHO) [9], all suggest that medical education should include integrated humanities and social sciences content and educators should adjust educational practices accordingly. These and other major health organizations increasingly argue for dental and medical education that prepares students to become ethical and humane doctors with professional integrity, a sense of social responsibility, leadership capacities, critical thinking and research competencies, and an orientation towards lifelong learning. Medical and dental curricula must therefore assist students in developing understandings of the causes of diseases, the distribution of healthcare benefits, the outcomes of healthcare practices, changes in medicine and dentistry, and socioeconomic, demographic, cultural, and individual factors in health [10–12].

To this end, MDHS courses have become a core part of the curriculum in many medical and dental schools, though the actual implementation of such courses comes with some difficulties. Foremost among these is the design of MDHS courses. Despite the efforts of educators to insert and improve the MDHS courses in their curriculum, no broad consensus exists about exactly what to teach future practitioners [13, 14]. The recent move to integrated courses covering the patient-doctor-society generally include content related to professionalism, policy, history, and research [15–17]. Nevertheless, little research has provided guidance for educators seeking to increase the effectiveness of MDHS course content design.

One difficulty concerns how to appropriately quantify the achievement of educational objectives and program effectiveness [18–22]. The most widely-used approach for measuring the effectiveness of MDHS courses has been to survey student satisfaction, a method that corresponds to Kirkpatrick’s basic level of educational program evaluation [23]. Yet, the educational standard for every curriculum should center on the graduates’ occupational competency in practice. According to Kirkpatrick, the effectiveness of education is best measured by the extent to which students transfer knowledge, skills, and attitudes from the classroom to the job, and further, adopt long-lasting aspirations to better their lives and professional environments [23].

Another difficulty is associated with the recognized status of the fields of humanities and social sciences by those in scientific fields. Many medical and dental students regard courses connecting the humanities-social sciences with health sciences to be clinically irrelevant, impractical, and even pointless [17, 24]. As a result, such courses occupy a lower status in the academic hierarchy than biomedical and clinical courses [13]. Furthermore, some students feel burdened or dissatisfied with the writing assignments such courses may require. This predisposition may result from a weaker grasp of fundamental concepts and methodologies in the humanities and social sciences compared to their proficiency in the biomedical and clinical sciences.

This study addresses the difficulties faced by medical and dental schools seeking to incorporate MDHS education into their curriculum. We investigated the design and implementation of an integrated MDHS course by measuring student growth in knowledge, attitudes, skills, and aspirations tied to the course outcomes as well as student evaluations of the significance of the course content. In order to generate knowledge that may assist in the effective design and implementation of MDHS curricula, we posed the following research questions:

1. To what extent did students exhibit gains in knowledge, attitudes, skills, and aspirations (KASA) following their participation in the integrated MDHS course? What growth differences exist among the four outcomes?
2. What is the relationship between student KASA growth and student achievement?
3. Which component(s) of the MDHS course did the students perceive as most important for their future careers? What, if any, differences exist among the components?
4. What is the relationship between student perceptions of the importance of course component(s) and KASA growth?

## Methods

### Integrated MDHS course design

Since there is no standard for best practices for an integrated MDHS course design, the course for this study, *Critical Understanding of Changing Dental Health Care*, was designed by classifying and synthesizing the standards and goals of medical and dental education proposed by the following seven international associations as shown in Table 1:

- ACGME - Accreditation Council for Graduate Medical Education, *Outcome Project*
- EC - European Commission, *The Tuning Project*
- GMC - General Medical Council, *Tomorrow’s Doctors*
- ADEA - American Dental Education Association, *Dentist’ Core Competency*
- ADEE - The Association for Dental Education in Europe, *Dentist’ Core Competency*
- WHO - World Health Organization, *Five Star Doctor*
- WFME - World Federation for Medical Education, *Role of the Doctor Project*

**Table 1:**
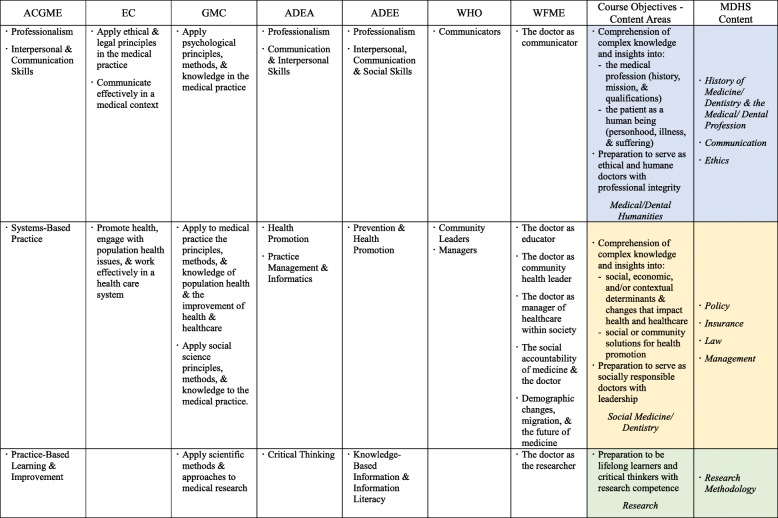
Competency standards or goals of medical/dental education and related MDHS content components

**Fig. 1:**
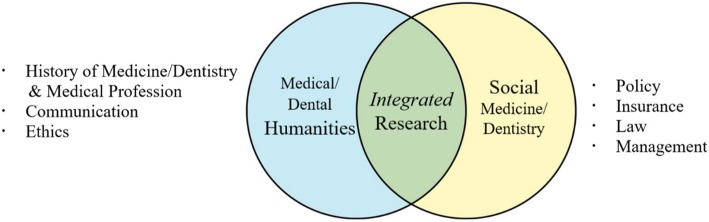
Integrated MDHS course components

The course was designed to incorporate, in an interrelated manner, content on medical/dental humanities, social medicine/dentistry, and integrated research as shown in Fig. 1. In 13 weeklong units, classes covered nine topics in the context of dentistry: history, professionalism, communication, ethics, management, policy, insurance, law, and research methodology (See Appendix 1). The aim of the course was to promote a complex and critical understanding of: (1) the medical profession (history, identity/mission, and qualifications of good doctors), (2) patients as humans (personhood, illness, and suffering), (3) social, economic, and contextual determinants that impact health and healthcare and related changes, (4) social and community solutions for health promotion, and (5) research methodologies. In a broader sense, the aim of the course was to prepare students to become ethical and humane doctors with professional integrity, social responsibility, leadership capacities, critical thinking and research competencies, and an orientation towards lifelong learning.

### Participants

Sixty-eight of seventy-five pre-doctoral students at a South Korean dental school gave written consent to participate in this study. Of the participants, 26.5% were female (*n* = 18) and 73.5% were male (*n* = 50); 70.6% were admitted under the 2 + 4 dental education system (*n* = 48) and 29.4% under the 4 + 4 system (*n* = 20). The average age of the participants was 25.2 (*SD* = 1.55). All were in the third year of their program and enrolled in the course *Critical Understanding of Changing Dental Health Care*. None had any previous exposure to an integrated MDHS course, but in the second year of their program all had participated in a course on medical communication.

### Data collection

Both student achievement data and survey data subsequently were collected as outcome measures for the study. Student achievement was defined in terms of performance on several in-class assessments: (1) three rounds of research paper development (40% of grade, weeks 4, 6, and 12), (2) two presentations (20% of grade, weeks 8 and 12), a final examination (30% of grade, following last class), and attendance and participation (10% of grade). The second author (JYL) collected the survey data from all 68 participants after the course final exam in December 2017. The response rate was 90.7% (68 out of 75).

The survey was composed of three parts and used a 5-point Likert scale. Part 1 of the survey asked for background data. Part 2 was comprised of 20 items designed to measure the students’ perceptions of their growth in four domains—knowledge, attitudes, skills, and aspirations (KASA)—following their participation in the course. These domains reflect Rockwell and Bennett’s (2004) well-established KASA framework for measuring educational outcomes. For each of the four domains, five questions were posed (See Appendix 2) [25]. Cronbach’s alphas were calculated for the internal consistency of the scales for each domain, resulting in 0.907 for knowledge, 0.896 for attitudes, 0.915 for skills, and 0.897 for aspirations. The internal consistency for the entire survey was 0.966. Part 3 of the survey asked students to rate the importance of each of nine course components—history, professionalism, communication, ethics, management, policy, insurance, law, and research methodology. Students were asked to rate the extent to which each of the components was of general importance (“I find it an important component of the course”), of importance to their future career (“I think that it is important to learn for my future career”), and of importance, but requiring more instruction (“I think that I need to learn more about it in the curriculum”). (See Appendix 3) Prior to collecting data, approval for the study (#2–2017-0053) was granted by the Institutional Review Board of the participating school.

### Data analyses

The data were analyzed using three types of statistical analysis. Descriptive statistics and within-subject one-way ANOVA were used with paired-samples t-tests as post-hoc comparisons in order to examine student perceptions about the effectiveness and significance of the integrated MDHS course. We conducted two sets of stepwise multiple regressions while conducting a preliminary test of the data to verify the assumptions of normality (histograms – symmetric bell-shaped, P-P plots), multi-collinearity (VIF < 10, Tolerance > 0.2) (Q3: VIF ranged 2.6~4.9, and Tolerance ranged 0.20~0.38 for knowledge, attitudes, skills and aspirations scales; Q4: VIF ranged 0.82~1.67, and Tolerance ranged 0.60~1.22), independence of error (*Dubin-Watson* value =2.00 for Q3, 1.64~2.13 for Q4), and homoscedasticity (scatter plot-random pattern).

## Results

### Q1. Growth in student KASA

First, we compiled the descriptive statistics and conducted a bivariate correlational analysis of the outcome variables. Overall, a high percentage of students reported growth in knowledge (83.8%), attitudes (77.6%), skills (82.6%), and aspirations (81.51%) following completion of the course. One-sample t-tests indicated that student responses were above neutral with large effect sizes (Cohen, 1988) (knowledge: M = 3.23, SD = 0.86, *t* (67) = 7.03, *d* = 0.85; attitudes: M = 3.14, SD = 0.83, *t* (67) = 6.41, *d* = 0.78; skills: M = 3.34, SD = 0.92, *t* (67) = 7.54, *d* = 0.91; aspirations: M = 3.24, SD = 0.87, *t* (67) = 7.02, *d* = 0.85). Students reported the greatest improvement in their skills followed by their aspirations, knowledge, and attitudes. Four outcome variables and final achievement were significantly and positively related to each other (knowledge-final achievement: *r* = 0.724, *p* = 0.000; attitudes-final achievement: *r* = 0.629, *p* = 0.000; skills-final achievement: *r* = 0.575, *p* = 0.000; aspirations-final achievement: *r* = 0.632, *p* = 0.000). A within-subject one-way ANOVA was conducted to investigate differences between each of the four KASA outcomes. Degrees of freedom was corrected using *Huynh-Feldt* estimates of sphericity (*ɛ* = 0.90) based on the result of a Manchly’s test (*χ*^2^ (5) = 15.15, *p* = 0.01). This result indicates a significant difference between the outcomes (*F* (2.69, 180.24) = 2.88, *p* = 0.045). Post-hoc analysis indicated no statistically significant difference in the growth of skills, aspirations, and knowledge, but a significant difference was observed in attitudes and skills (*t* (67) = − 2.604, *p* = 0.011, *d* = 0.23). Attitude growth showed the smallest change (*M* = 15.71, *SD* = 4.13) while skills growth showed the largest (*M* = 16.71, *SD* = 4.60).

### Q2. The relationship between KASA growth and course achievement

We conducted multiple regressions to investigate the relationships between student growth in knowledge, attitudes, skills, and aspirations and course achievement. We found a significant regression eq. (*F* (4, 63) = 17.700, *p* < 0.000), with an *R*^*2*^ of 0.529 (*Adj. R*^*2*^ = 0.499). We found knowledge growth to be the only significant explanatory variable (*β* = 0.635, *t* (63) = 3.394*, p* = 0.001). According to Cohen’s guidelines, the relationship between perceived objective attainment and knowledge growth has a large effect size (*f*^*2*^ = 1.123).

### Q3. Perceived importance of course components for future career

A within-subject one-way ANOVA was conducted to investigate differences between student satisfaction with components of the course content. We corrected degrees of freedom using *Greenhouse-Geisser* estimates of sphericity (*ɛ* = 0.63) based on Manchly’s test (*χ*^2^ (20) = 92.14, *p* = 0.00). The results show a significant difference in student satisfaction with different course components (*F* (3.76, 251.64) = 19.31, *p* = 0.00). Students perceived the importance of content on insurance to be the most critical content component, followed by policy, management, and law. Students rated the importance of content on professionalism as least important for their future career. We found no significant differences between aspects of the humanistic content (history, professionalism, ethics, and communication) or between aspects of the social sciences content (insurance, policy, management, and law). Significant differences were found in the perceived importance of various aspects of the humanistic content and social sciences content, with effect sizes *d* ranging from 0.594 to 0.868, indicating large effects. In general, students tended to regard the social sciences content as more important than the humanistic content.

### Q4. The relationship between student perceptions of the significance of course components and KASA growth

We conducted stepwise multiple regressions to explore the relationships between student perceptions of the significance of the course content components and the outcome variables (Table 2). The perceived significance of research was the common significant explanatory variable for all growth in KASA and the total outcome. The perceived importance of research (*β* = .644, *t* (63) = 6.659, *p* = 0.000) and professionalism (*β* = 0.213, *t* (63) = 2.203, *p* = 0.031) were significantly related to knowledge growth; in the final model these two variables explained 63.4% of the variances in knowledge growth. Regarding growth in attitudes and skills, the perceived importance of research was the only significant explanatory variable for growth in attitudes (*β* = 0.784, *t* (63) = 10.257, *p* = 0.000) and in skills (*β* = 0.769, *t* (63) = 9.772, *p* = 0.000); it explained 61.4% of the variances in attitude growth and 59.1% in skill growth. Growth in aspirations was significantly related to the perceived importance of research (*β* = 0.639, *t* (63) = 7.595, *p* = 0.000) and law (*β* = 0.265, *t* (63) = 3.145, *p* = 0.003); the final model suggested that these two variables explained 62.3% of the variances in aspirational growth. The composite variable of all the outcome variables was explained by the perceived importance of research (*β* = 0.792, *t* (63) = 11.391, *p* = 0.000) and policy (*β* = 0.140, *t* (63) = 2.017, *p* = 0.048), with an explanatory power of 72.2%.

**Table 2:** Regressions for outcome variables

Dependent Variables	Explanatory Variables	*b*	*SE b*	*B*	*T*	*P*	*R*^*2*^ (adj. *R*^*2*^)	*F*
Knowledge	Research	3.008	.452	.644	6.659	.000	.634 (.623)	56.273^***^
	Professionalism	.987	.448	.213	2.203	.031		
Attitudes	Research	3.531	.344	.784	10.257	.000	.614 (.609)	105.203^***^
Skills	Research	3.860	.395	.769	9.772	.000	.591 (.585)	95.483^***^
Aspirations	Research	3.039	.400	.639	7.598	.000	.623 (.612)	53.784^***^
	Law	1.518	.483	.265	3.145	.003		
Final Achievement	Research	2.756	.242	.792	11.391	.000	.722 (.713)	84.225^***^
	Policy	.557	.276	.140	2.017	.048		

^***^p < .001

## Discussion

Our aim in this study was to explore the potential effects of an integrated MDHS course on student course achievement and growth in knowledge, attitudes, skills, and aspirations. We also investigated student perceptions of the importance of the course content and the relationship of these perceptions to course achievement and growth in KASA.

A majority of students (77.6%~ 83.8%) reported positive growth following their participation in the MDHS course. Students reported the greatest growth in skills, followed by aspirations, knowledge, and attitudes, in that order. Skill growth referred mainly to how familiar students had become with methods for conducting research. Further, compared to their behavior in biomedical or clinical science courses, students in the MDHS course spent more time collecting and summarizing the materials needed to answer their research question, constructing a suitable theory, and deriving conclusions. The experience of writing up their research findings may account for their strong sense that they had gained important skills [26]. We found the substantial growth in aspirations reported by students to be very encouraging. Even after completing the course, students aspired to participate in more training in related areas and engage in efforts to further their development.

Professional competency is composed of practical wisdom acquired through extensive experience, along with ongoing professional development and evolving knowledge and skills [27]. The third greatest gains reported by the participants were in knowledge, and only students who perceived growth in knowledge remarked that the course was organized suitably and had achieved its stated goals. Since most medical and dental courses are composed of lectures and written examinations, there is a danger that students may believe that skills acquired through project activities or the growth of aspirations are not directly relevant to accomplishing the course objectives.

Student perceptions about the importance of course content may correspond to their perceived future professional needs as well. Students recognized the importance of knowledge about core aspects of the public healthcare system, such as insurance, policy, management, and law. They tended to value content within the social sciences more than content in the medical humanities. Although students are aware that they need to be humanistic doctors in the long run [17], they seemed to feel that the practical issues they may face as future practitioners are addressed more directly by the content found in the social sciences. They expressed the need for coursework that covers practical knowledge and action guidelines for specific subjects. Another reason for greater valuing of social science content may be that the discipline of medicine shares more with the social sciences from the perspective of methodology and scholarly analysis, such as the use of deductive reasoning, hypotheses, and evidence, elements of the scientific method with which the participants may have been more familiar.

We may consider why the perceived importance of professionalism was significantly related to knowledge growth. First, as students learned about the history of the medical professions it may have led them to consider their own professional identity. Such self-conceptions may play an important role in their relationships with patients and colleagues, as well as in their own sense of wellbeing. Second, as the students more seriously considered their roles as professionals, their motivation to acquire more knowledge about current health management systems may have grown. Finally, as they conducted their research projects, students were able to apply the core values of professionalism as they sought to critically understand changes in the healthcare system and envision concrete courses of action.

One of the most noteworthy findings was that students who more strongly perceived the integrated research activity as important exhibited the most marked growth in all KASA aspects. Student perceptions about the importance of the research component of the course may be an indicator of how well the MDHS course integrated the humanities and social sciences in the students’ minds, persuading them of the need for such an MDHS course. By conducting research activities, students practiced activities that engaged their critical thinking skills as they sought to resolve complex real-life issues related to dental care [28–30]. Engagement in critical thinking can provoke students’ multi-disciplinary contemplation and reflection when facing complex issues in medicine and dentistry that are often characterized by ambiguity and uncertainty [13, 31, 32].

## Conclusions

The proposed MDHS course that served as the focus of this study covered three major areas: medical humanities (history and professional identity of medical professionals, communication, and ethics), social medicine (policy, insurance, legal dispute, and management), and integrated research. We found that an MDHS course needs to demonstrate how the topics in the humanities and social sciences are related to each other. In addition, the design needs to help students assimilate such topics into their integrated research. In this way, students in the health sciences may become convinced of the importance of both humanities and social sciences, actively engaged in learning activities for attitudinal growth, and assessed their growth in balanced perspectives.

This study has a number of limitations. First, it was conducted under a cross-sectional design. More long-term and dynamic tracking of the learning outcomes as students grow to be medical professionals would be desirable, suggesting a need for a well-designed longitudinal investigation. Although student self-assessment (SSA) has been known to be valid for particular participants and research contexts [33–35] (to which this study conformed) and the KASA instrument has been used widely, the use of SSA as the primary source of data entails limitations. Future studies, then, should adopt pre- and post-measures to better assess actual changes in KASA. Additionally, more research is required to determine whether research activities actually do yield improvement in critical thinking and whether the perceived learning outcomes persist in the long term [36].

Because the great majority of participants were male, this study may not be generalizable to all medical and dental programs. Further research needs to be conducted in order to extend these results to a gender-balanced population. Lastly, although the regression models in this study showed fairly strong associations between dependent variables and explanatory variables, the totality of evidence is insufficient to generalize about causal relationships. Further validation is required using new datasets, and ideally, a large scale RCT. Nevertheless, we hope that this study will contribute to the improved design of MDHS courses in order to strengthen medical practitioners’ professional competencies and improve the public health.

## Supplementary information

**Additional file 1. **Appendix 1. Weekly plan for course content. Appendix 2. KASA Measurement Items. Appendix 3. Students’ Need Survey Items

## Data Availability

Related data from this study is available upon request from the second author.

## Abbreviations

ACGME: Accreditation Council for Graduate Medical Education
ADEA: American Dental Education Association
ADEE: The Association for Dental Education in Europe
ANOVA: Analysis of Variance
EC: European Commission
GMC: General Medical Council
KASA: Attitudes, Attitudes, Skills, and Aspirations
MDHS: Medical/Dental Humanities-Social medicine/dentistry
RCT: Randomized Controlled Trial
WFME: World Federation for Medical Education,
WHO: World Health Organization

## References

[1] Donohoe M, Danielson S (2004). A community-based approach to the medical humanities. Med Edu..

[2] Doukas DJ, McCullough LB, Wear S (2010). Reforming medical education in ethics and humanities by finding common ground with Abraham Flexner. Acad Med.

[3] Krackov SK, Levin RI, Catanese V, Rey M, Aull F, Blagev D (2003). Medical humanities at New York University School of Medicine: an array of rich programs in diverse settings. Acad Med.

[4] Jones T, Blackie M, Garden R, Wear D (2017). The almost right word: the move from medical to health humanities. Acad Med.

[5] Hall JN, Woods N, Hanson MD (2014). Is social sciences and humanities (SSH) premedical education marginalized in the medical school admission process? A review and contextualization of the literature. Acad Med.

[6] Rourke J. Social Accountability: A framework for medical schools to improve the health of the populations they serve. Acad Med*.* 9000;Publish Ahead of Print. 10.1097/ACM.0000000000002239 29642103

[7] General Medical Council. Tomorrow’s doctors: Outcomes and standards for undergraduate medical education. London, UK: General Medical Council (GMC); 2009.

[8] Gordon D, Lindgren SC. The global role of the doctor in healthcare. World Medical & Health Policy*.* 2012; *2*(1):[19–29 pp.]. Available from: http://wfme.org/home/projects/role-of-the-doctor/.

[9] Boelen C. The five-star doctor: an asset to health care reform. Human Resources Development Journal*.* 2001; 1(1). Available from: http://www.who.int/hrh/en/HRDJ_1_1_02.pdf?ua=1.

[10] Kasper J, Greene JA, Farmer PE, Jones DS (2016). All health is global health, all medicine is social medicine: integrating the social sciences into the preclinical curriculum. Acad Med.

[11] Lindgren S, Gordon D (2011). The doctor we are educating for a future global role in health care. Med Teach.

[12] Westerhaus M, Finnegan A, Haidar M, Kleinman A, Mukherjee J, Farmer P (2015). The necessity of social medicine in medical education. Acad Med.

[13] Kumagai AK (2017). Beyond “Dr. feel-good”: a role for the humanities in medical education. Acad Med.

[14] Siegel J, Coleman DL, James T (2018). Integrating social determinants of health into graduate medical education: a call for action. Acad Med.

[15] Branch WT (2005). Use of critical incident reports in medical education. A perspective. J Gen Intern Med.

[16] Lee J (2016). A study on a qualitative research paper and essay done in dental class. J Korean Dent Assoc.

[17] Shapiro J, Coulehan J, Wear D, Montello M (2009). Medical humanities and their discontents: definitions, critiques, and implications. Acad Med.

[18] Doukas DJ, Kirch DG, Brigham TP, Barzansky BM, Wear S, Carrese JA (2015). Transforming educational accountability in medical ethics and humanities education toward professionalism. Acad Med.

[19] Doukas DJ, McCullough LB, Wear S (2012). Perspective: medical education in medical ethics and humanities as the foundation for developing medical professionalism. Acad Med.

[20] Ousager J, Johannessen H (2010). Humanities in undergraduate medical education: a literature review. Acad Med.

[21] Wear D, Zarconi J (2008). Can compassion be taught? Let's ask our students. J Gen Intern Med.

[22] Schwandt TA (1988). Evaluating the medical humanities. Teach Learn Med..

[23] Kirkpatrick DL, Kirkpatrick JD (2006). Evaluating training programs: the four kevels.

[24] Greene JA, Jones DS (2017). The shared goals and distinct strengths of the medical humanities: can the sum of the parts be greater than the whole?. Acad Med.

[25] Rockwell SK, Bennett CF. Targeting outcomes of programs (TOP): a hierarchy for targeting outcomes and evaluating their achievement 2004. Available from: http://digitalcommons.unl.edu/aglecfacpub/48.

[26] Saffran L (2017). Emotional life: exploring contradictions in health behavior through creative writing in public health education. Acad Med.

[27] Mann KV (2011). Theoretical perspectives in medical education: past experience and future possibilities. Med Edu.

[28] Krupat E, Sprague JM, Wolpaw D, Haidet P, Hatem D, O'Brien B (2011). Thinking critically about critical thinking: ability, disposition or both?. Med Edu.

[29] Tiwari A, Lai P, So M, Yuen K (2006). A comparison of the effects of problem-based learning and lecturing on the development of students' critical thinking. Med Edu.

[30] Friedman LD (2002). The precarious position of the medical humanities in the medical school curriculum. Acad Med.

[31] Ofri D (2017). Medical humanities: the Rx for uncertainty?. Acad Med.

[32] Polianski IJ, Fangerau H (2012). Toward “harder” medical humanities: moving beyond the “two cultures” dichotomy. Acad Med.

[33] Brown GT, Andrade H, Chen F (2015). Accuracy in student self-assessment: directions and cautions for research. Assess Educ.

[34] Ross JA (2006). The reliability, validity and utility of self-assessment. Pract Assess Res Eval.

[35] Torres MB, Chochran A (2016). Accuracy and content of medical student midclearkship self-evaluation. Am J Surg.

[36] Rabow MW, Lapedis M, Feingold A, Thomas M, Remen RN (2016). Insisting on the healer's art: the implications of required participation in a medical school course on values and humanism. Teach Learn Med.

