# Supplementary material for: An integrated humanities–social sciences course in health sciences education: proposed design, effectiveness, and associated factors
Source: BMC Med Educ. 2020 Apr 19;20:117. doi: 10.1186/s12909-020-02022-7 (PMC7168810; doi:10.1186/s12909-020-02022-7)
Supplement: Supplementary file 1 — Additional file 1. Appendix 1. Weekly plan for course content. Appendix 2. KASA Measurement Items. Appendix 3. Students’ Need Survey Items [file 12909_2020_2022_MOESM1_ESM.docx]

**Appendix 1.** Weekly plan for course content

|  | Course Components | Sub-Topics |
| --- | --- | --- |
| Week 1 | History  Research Methodology | - History of dentistry and dental profession - Goals of research in medicine/dentistry, Lifelong learning - Qualitative research methodology |
| Week 2 | History  Professionalism | - Changes in roles of dentists/health professionals throughout history - The mission of (oral) healthcare - History of health/dental care system worldwide |
| Week 3 | Management | - Management and medical management - Medical socialization - Social or community solutions for (oral) health promotion, Socially responsible doctors with leadership - Future of medical management |
| Week 4 | Insurance  Research Methodology | - Historical changes in the Korean health insurance - Issues and future directions for dental health insurance - Quantitative research methodology |
| Week 5 | Law | - Historical and recent changes in Korean dental care laws and legal regulations - Medical laws of other countries - Major issues in medical law |
| Week 6 | Policy | - History and recent issues in Korean dental health policy - Social, economic, or contextual determinants of oral health and healthcare - Future directions for dental health policy - The role of dentists in policy proposals and implementation |
| Week 7 | Law | - Legal disputes - The causes of the disputes - Patterns, countermeasures, and prevention in regard to disputes |
| Week 8 | Midterm Research Presentation | |
| Week 9 | Ethics | - Philosophical understanding of the health sciences profession - Conceptions of normality and abnormality - Ethical approaches to science |
| Week 10 | Communication | - Patient-doctor clinical communication - The patient as a human being (personhood, illness, & suffering) - Inter-professional communication |
| Week 11 | Professionalism | - The future of health care worldwide - New approaches and paradigms of professionalism for a changing society |
| Week 12 | Final Research Presentation | |
| Week 13 | Final Test | |

**Appendix 2.** KASA Measurement Items

| KASA | Survey items |
| --- | --- |
| Knowledge  increased awareness, understanding, and/or problem solving ability | 1. I gained new knowledge about the dental care system in this course. 2. I have come to understand the current and historical flow of dental care in this course. 3. I gained knowledge of research in the field of DHS in this course. 4. I learned how to think about solving problems in this field. 5. I can apply the knowledge gained from this course to actual dental/medical situations. |
| Attitudes  changed outlooks, perspectives, or viewpoints | 1. I was curious about each subject in this course. 2. This course has broadened my perspective on each topic. 3. I came to think that the topics covered in this course were more significant than before. 4. I think I can adopt a more professional attitude toward related topics. 5. I became more active in designing the future rather than worrying about change. |
| Skills  improved verbal, cognitive, or physical abilities for better performance | 1. I learned how to find statistical data and articles related to this field. 2. I learned how to synthesize references in the field. 3. I learned about the process and skills for writing research papers in this field. 4. I learned about research methodologies, such as comparative research, critical understanding, issue analysis, and judgment. 5. I learned how to interpret issues related to this field. |
| Aspirations  altered desires, ambitions, hopes, or behaviors | 1. I will continue to search for materials related to this field. 2. I will find and read papers in this field when I need them. 3. I will continue to communicate with my colleagues on these topics. 4. I would like to know more about the expertise of the instructors who participated in the course. 5. I want to utilize what I learned in this course to become a successful dentist. |

**Appendix 3.** Students’ Need Survey Items

Please rate the importance of each course component from 1 (not at all important) to 5 (very important).

|  | General importance^1^ | Importance for future career^2^ | Need to expand within curriculum^3^ |
| --- | --- | --- | --- |
| History | ( 5 / 4 / 3 / 2 / 1) | ( 5 / 4 / 3 / 2 / 1) | ( 5 / 4 / 3 / 2 / 1) |
| Professionalism | ( 5 / 4 / 3 / 2 / 1) | ( 5 / 4 / 3 / 2 / 1) | ( 5 / 4 / 3 / 2 / 1) |
| Communication | ( 5 / 4 / 3 / 2 / 1) | ( 5 / 4 / 3 / 2 / 1) | ( 5 / 4 / 3 / 2 / 1) |
| Ethics | ( 5 / 4 / 3 / 2 / 1) | ( 5 / 4 / 3 / 2 / 1) | ( 5 / 4 / 3 / 2 / 1) |
| Management | ( 5 / 4 / 3 / 2 / 1) | ( 5 / 4 / 3 / 2 / 1) | ( 5 / 4 / 3 / 2 / 1) |
| Policy | ( 5 / 4 / 3 / 2 / 1) | ( 5 / 4 / 3 / 2 / 1) | ( 5 / 4 / 3 / 2 / 1) |
| Insurance | ( 5 / 4 / 3 / 2 / 1) | ( 5 / 4 / 3 / 2 / 1) | ( 5 / 4 / 3 / 2 / 1) |
| Law | ( 5 / 4 / 3 / 2 / 1) | ( 5 / 4 / 3 / 2 / 1) | ( 5 / 4 / 3 / 2 / 1) |
| Research methodology | ( 5 / 4 / 3 / 2 / 1) | ( 5 / 4 / 3 / 2 / 1) | ( 5 / 4 / 3 / 2 / 1) |

^1^ I find _______ to be an important component of the course.

^2^ I think that it is important to learn about _______ for my future career.

^3^ I think that I needed to learn more about _______ in the curriculum.
